# Characterization and therapeutic evaluation of the lytic bacteriophage ENP2309 against vancomycin-resistant *Enterococcus faecalis* infections in a mice model

**DOI:** 10.1186/s12985-025-02917-1

**Published:** 2025-08-28

**Authors:** Jiaqi Tian, Luyao Wang, Rui Gao, Wenwen Zhou, Shinan Zhang, Lingxia Li, Guoyuan Hu, Licheng Xiao, Yijuan Ma, Sang Ba, Daijiyongzang, Shengyi Han, Shengqing Li

**Affiliations:** 1 Qinghai Academy of Animal Sciences and Veterinary Medicine, Qinghai University, Xining, 810016 China; 2 College of Agriculture and Animal Husbandry, Qinghai University, Xining, 810016 China; 3 Qinghai Provincial Key Laboratory of Pathogen Diagnosis for Animal Disease and Green, Technical Research for Prevention and Control, Xining, 810016 China; 4 Animal husbandry and veterinary workstation of Yushu City, Yushu, 815000 China

**Keywords:** Bacteriophage, VRE, Biological characteristics, Genome, Therapy

## Abstract

**Background:**

The global emergence of vancomycin-resistant *Enterococci* (VRE) represents a growing threat to public health worldwide. To address this critical challenge, we isolated and characterized a novel lytic bacteriophage, ENP2309, from agricultural wastewater. Comprehensive analysis revealed distinct morphological features, biological properties, and genomic characteristics of ENP2309. Most notably, systematic evaluation in a mice infection model demonstrated significant in vivo therapeutic efficacy.

**Methods:**

Bacteriophage isolation was performed using the double-layer agar method with the *Enterococcus faecalis* strain. Phage morphology was characterized by transmission electron microscopy (TEM), The host range was determined via plaque assays and the plating efficiency of multiple bacterial isolates. was evaluated double-layer agar method was systematically employed to evaluate thermal stability, pH tolerance, one-step growth kinetics, and the optimal multiplicity of infection (MOI) through plaque-forming unit (PFU) enumeration. The genomic features were analysed using next-generation sequencing. Furthermore, the therapeutic efficacy of phage ENP2309 against Enterococcal infection in mice was systematically evaluated through a comprehensive assessment of multiple parameters including body weight dynamics, survival rates, histopathological analysis, peripheral blood cytokine profiles, and bacterial loads in the spleen and liver tissues, demonstrating its multidimensional therapeutic effects.

**Results:**

The phage ENP2309 showed broad-spectrum lytic capability, effectively targeting 13 distinct Enterococcus clinical isolates. TEM revealed the morphology of ENP2309, featuring an icosahedral capsid (70 ± 1 nm in diameter) and a contractile tail structure (145 ± 2 nm in length). Comprehensive biological characterization revealed optimal infection parameters including an exceptionally low multiplicity of infection (MOI = 0.001), a 40-minute latent period, and an extended 40–120 min burst period resulting in a burst size of 920 PFU/cell. The phage exhibited environmental stability, maintaining infectivity across broad temperature (10–60 °C) and pH (3–12) ranges, with optimal activity observed at 37 °C and neutral pH (7.0–7.5). Genomic analysis revealed a 148,806 bp linear dsDNA (35.9% GC content) containing 153 putative ORFs. Phylogenetic classification revealed that ENP2309 a member of the *Kochikohdavirus* genus, is closely related to the Enterococcus phage PBEF129. In vivo studies demonstrated exceptional therapeutic potential: a single dose (200 µL 2.0 × 10⁸ PFU/mL) of phage ENP2309 achieved 100% survival in mice models, completely clearing VRE from the spleen and liver while significantly improving physiological parameters, reducing organ damage, and attenuating systemic inflammation.

**Conclusions:**

These comprehensive findings establish ENP2309 as a highly promising therapeutic alternative to conventional antibiotics for VRE infections with distinct advantages.

## Introduction

The antibiotic revolution has triggered an ancient microbial defence system, with genomic evidence revealing antimicrobial resistance mechanisms existing millennia before clinical antibiotic use [1–2]. Antimicrobial-resistant pathogens now claim 700,000 lives annually and threaten 10 million deaths/year by 2050 without intervention [3]. Vancomycin-resistant *Enterococcus* (VRE), a high-priority pathogen, has demonstrated alarming adaptability through three evolutionary strategies: intrinsic β-lactam resistance, acquired vancomycin resistance, and mobile virulence factors, enabling lethal infections such as endocarditis septicemia and meningitis [4–5]. The crisis has escalated as > 80% of *Enterococcus* clinical isolates develop multidrug resistance spanning vancomycin, β-lactams, fluoroquinolones, and tetracyclines [6–7]. Global surveillance reveals accelerating resistance rates from 30% in U.S. health care infections to European hotspots exceeding 66% (Lithuania), necessitating the urgent development of next-generation antimicrobials [8–9].

Bacteriophages, Earth’s most abundant biological entities, were first recognized for their therapeutic potential by William Twort in 1915 [10], leading to widespread clinical use in Eastern Europe by the 1920s before being overshadowed by broad-spectrum antibiotics in the mid-20th century [11]. Their current resurgence responds to the MDR crisis, offering unique advantages: species-specific targeting that preserves the commensal microbiota, self-amplification at infection sites enabling reduced dosing, and environmental ubiquity permitting rapid isolation from wastewater/soil reservoirs. These natural predators additionally penetrate biofilms and coevolve with bacterial resistance, overcoming key limitations of conventional antibiotics while maintaining gut ecological balance—critical attributes positioning phage therapy as a precision weapon against resistant infections. Despite the numerous advantages of bacteriophages over to antibiotics, the limitations of phage therapy remain significant. As antigens, bacteriophages can elicit an immune response in the host. Unlike antibiotics, bacteriophages possess a narrow antibacterial spectrum. This specificity implies that, for example, *Escherichia coli* infections require the application of *Escherichia coli*-specific phages, and *Enterococcus* infections necessitate *Enterococcus-*specific phages. Consequently, prior to phage administration, the pathogenic bacteria must be isolated from the patient and matched with appropriate phages. This requirement renders phage therapy less convenient than antibiotic therapy. Like antibiotics, bacteria can also develop resistance to bacteriophages [12]. To advance phage therapy towards clinical application, a systematic research framework must be established to: fully characterize phage biological properties and genetic determinants to ensure therapeutic efficacy and safety; establish rigorous selection criteria requiring strictly lytic phages with broad-spectrum activity against target pathogens; and implement comprehensive genomic screening to eliminate strains harbouring virulence, resistance, or pathogenicity factors. This multifaceted approach will enable the optimization of phage therapeutics while mitigating potential clinical risks.

In this study, we isolated and characterized ENP2309, a novel lytic Enterococcus phage, from farm sewage. Comprehensive analysis revealed its distinct morphological characteristics, biological properties, and genomic organization. Notably, ENP2309 exhibited 100% protective efficacy against VRE infections in mice, positioning it as a promising therapeutic candidate for combating VRE infections in clinical practice.

## Materials and methods

### Phage isolation and purification

This study utilized 19 strains of *Enterococcus* from the Key Laboratory of Animal Disease Pathogen Diagnosis and Green Prevention and Control Technology in Qinghai Province as host bacteria. Wastewater samples were collected from various yak farms in Xining city for phage isolation. The phage isolation methods used were previously described by Liu et al. [13], and the phage isolation and purification process was as follows:

First, wastewater samples were sterilized by filtration through a 0.22 μm membrane filter (Millipore). The filtrate was then mixed with the host bacteria at a 1:1 ratio (v/v). The mixture was then plated using the double-layer agar method (the bottom layer was 1.5% agar-solidified Todd-Hewitt broth, and the top layer was THB medium containing 0.5% agar). After incubation at 37 ℃ overnight, the formation and morphology of phage plaques were observed. A single phage plaque was subsequently aseptically picked and suspended in sterile PBS at room temperature for 4 h to allow phage dissociation. This suspension was then mixed again with the host bacteria and replated using the double-layer agar method. After overnight incubation at 37 °C, single plaques were isolated. This purification process was repeated three times to ultimately obtain purified phage isolates.

### Host range

Suspensions of distinct *Enterococcus* strains were spread onto double-layer agar plates and preincubated at 37 °C for 2 h to form bacterial lawns. Subsequently, 5 µL aliquots of phage solution were spotted onto the solidified agar surface. Following overnight incubation at 37 °C, lytic activity was evaluated by counting the infection classes (+ 4, + 3, +2, + 1, and 0) within the bacterial lawns (Table 1). The methods described by Fayez et al. [14] were subsequently followed, *Enterococcus faecalis* GZ25 was used as the indicator bacterium, the efficiency of plating (EOP) was calculated. The EOP is defined as the ratio of the lysis titre of the phage against different test strains to that against the indicator bacterium.

**Table 1:** Scoring criteria for plaque Lysis activity

Score	Degree of Lysis	Morphological Description
**+ 4**	**Complete lysis**	The spotted area is entirely clear with no visible bacterial lawn residue, forming a distinct plaque (appearing “hollow”).
**+ 3**	**Significant lysis**	The spotted area is mostly clear, with slight bacterial lawn residue at the edges; plaque boundaries are well-defined.
**+ 2**	**Partial lysis**	The spotted area is semi-clear, with scattered unlysed colonies or a hazy bacterial lawn; plaque boundaries are indistinct.
**+ 1**	**Weak lysis**	Only small localized clear zones (e.g., pinprick-sized plaques) are present; the bacterial lawn remains largely intact and requires close observation to detect lysis.
**0**	**No lysis**	The bacterial lawn fully covers the area with no visible clearing or signs of lysis.

### Morphological observation

The phage particles were sequentially concentrated using PEG 8000 (Sigma‒Aldrich) precipitation followed by CsCl density gradient centrifugation according to established protocols [15]. The purified phage suspension was adsorbed onto 400-mesh carbon-coated copper grids (Ted Pella, USA) and stained with 1% (w/v) phosphotungstic acid (SolarBio, Beijing, China) for 10 min at room temperature. The grids were air-dried and subsequently imaged using an HT7700 transmission electron microscope (Hitachi, Japan) at 80 kV.

### Temperature stability

Phage ENP2309 suspensions were subjected to thermal stability testing through 1-hour incubation at incremental temperatures ranging from 10 °C to 80 °C (10 °C intervals). Following thermal treatment, residual phage viability was quantified via the double-layer agar method. All experimental conditions were evaluated through triplicate independent biological replicates.

### pH stability

The pH of the SM buffer was adjusted to values ranging from 2 to 13 using concentrated hydrochloric acid or sodium hydroxide solution. A total of 100 µL of phage mixture was added to 900 µL of SM buffer at various pH values and allowed to stand at 37 °C for 1 h. The phage titres were determined using the double-layer method. All experimental conditions were evaluated through triplicate independent biological replicates.

### Optimal multiplicity of infection

Serial dilutions of phage ENP2309 were combined with host bacterial cultures (OD_600_ = 0.6) under conditions of varying multiplicity of infection (MOI = 10^− 5^, 10^− 4^, 10^− 3^, 10^− 2^, 10^− 1^, 1, 10, 10^2^, 10^3^, 10^4^, and 10^5^). After 10 min adsorption at 37 °C, the unbound phages were removed by centrifugation (8,000 r/min, 10 min, 4°C). The bacterial pellets were resuspended in 10 mL of fresh THB broth and incubated at 37 °C, and 200 r/min for 4 h. Phage progeny production was quantified through the double-layer agar method, The experiment was performed in triplicate.

### One step growth

In accordance with on the methods of Zurabov et al. [16], with adjustments made for this study, the following protocol was used: host bacterial cultures (OD_600_ = 0.6) were infected with phage ENP2309 at an MOI of 0.001. A mixture of 100 µL of phage suspension and 100 µL of host bacterial culture was incubated at 37 °C for 10 min. The mixture was then centrifuged (8,000 r/min, 5 min, 4 °C). The resulting pellet was collected and resuspended in 50 mL of THB broth. The mixture was incubated in a shaking incubator (37 °C, 200 rpm/min). Every 20 min, a 2 mL aliquot of the suspension was collected, and the phage suspension was obtained by filtration through a 0.22 μm filter. Phage titres were determined using the double-layer agar plate method, and monitored continuously for 6 h. The latent period was defined as the time required for phage adsorption to the host cell and subsequent release of progeny phages. The burst size of the phage was expressed as the ratio of the final count of phage particles released during the outburst to the number of infected bacterial cells. Burst size = (Peak phage titer) / (Total number of infected bacteria in the system). The experiment was performed in triplicate.

### Phage genome extraction and sequencing

The phage genome was extracted using a bacteriophage DNA isolation kit (Norgen Biotek, Canada) following the manufacturer’s protocol. The purified DNA was quantified using a Nano Drop One (Thermo Fisher Scientific) and assessed for quality by agarose gel electrophoresis (1% w/v). Sequencing libraries were prepared using the Illumina TruSeq Nano DNA LT Library Prep Kit (Illumina, San Diego, CA) according to the manufacturer’s instructions (Illumina TruSeq DNA Sample Preparation Guide, Rev. E) at Shanghai Paisenno Bio-Tech Co., Ltd. Raw sequencing reads were quality-filtered using Trimmomatic (v0.39) and assembled de novo using SPAdes (v3.12.0) with default parameters. Contigs were screened on the basis of sequencing depth (> 50× coverage), and high-depth sequences were subjected to BLASTn analysis against the NCBI NT database [17] for phage genome identification. The final complete genome sequence was validated through manual curation and comparison with reference phage genomes in the NCBI database.

### Phage genome analysis

The online BLASTp server was used to identify open reading frames (ORFs). The online tools ResFinder (http://genepi.food.dtu.dk/resfinder) and VirulenceFinder (https://cge.cbs.dtu.dk/services/) were used to screen for antibiotic resistance genes and virulence genes in the phage genome. tRNAs were predicted using tRNA scan SE (http://lowelab.ucsc.edu/tRNAscan-SE/index.html). A phage genome map was generated using SnapGene 6.0.2. To analyse the phylogenetic relationships of the phage, the genome sequence was used for a BLASTn search of the NCBI database. A phylogenetic tree based on the large terminase subunits of related phages was constructed by the neighbour‒joining method in MEGA 7.0 [18]. Phage genomes were compared using VIRIDIC [19] to determine their genetic relatedness (≥ 70% nucleotide sequence identity implies a genus-level relationship, whereas ≥ 95% identity suggests a species-level relationship).

### Evaluation of phage therapeutic effects

Female BALB/C mice (16–18 g, purchased from the Lanzhou Institute of Animal Husbandry and Veterinary Medicine) were randomly allocated into four experimental groups (*n* = 10 per group): (a) Challenge group: Mice were intraperitoneally challenged with *E. faecalis* GZ16185 at the minimum lethal dose (3.0 × 10¹¹ CFU/mL). (b) Treatment group: 200 µL of phage ENP2309 (2.0 × 10⁸ PFU/mL) was administered via intraperitoneal injection 15 min post-infection. (c) Phage group: This group received 200 µL of phage ENP2309 alone (2.0 × 10⁸ PFU/mL) intraperitoneally. (d) PBS group: Injected intraperitoneally with 200 µL of PBS buffer.

The experimental cycle spanned 14 days. Throughout the study, daily body weight, survival rates, and general health status were recorded for all groups.

In addition to the mice in the challenge group (Day 3), the mice in the other groups were assessed at the endpoint (Day 14). The mice were euthanized, and the organs (e.g., liver, and spleen) were harvested for histopathological evaluation. Tissue sections were prepared and stained with haematoxylin-eosin (HE) to assess pathological changes. The bacterial loads in the spleen and liver were quantified using the plate counting method.

Blood samples were collected via retro-orbital bleeding on Days1, 3, 5, and 7 posttreatment. Serum cytokine levels (e.g., TNF-α, IL-6 and IL-10) were quantified using commercial ELISA kits according to the manufacturers’ protocols.

### Statistical analysis

All data were processed for statistical analysis (means and standard deviations) using SPSS 22.0, and graphical representations were generated using OriginPro 2021. Group differences were evaluated by one-way ANOVA, followed by Bonferroni correction. *P* values < 0.05 were considered statistically significant. Significance is indicated in the figures by asterisks (*, *P* < 0.05; **, *P* < 0.01; ***, *P* < 0.001).

## Results

### Isolation and microscopy

**Fig. 1:**
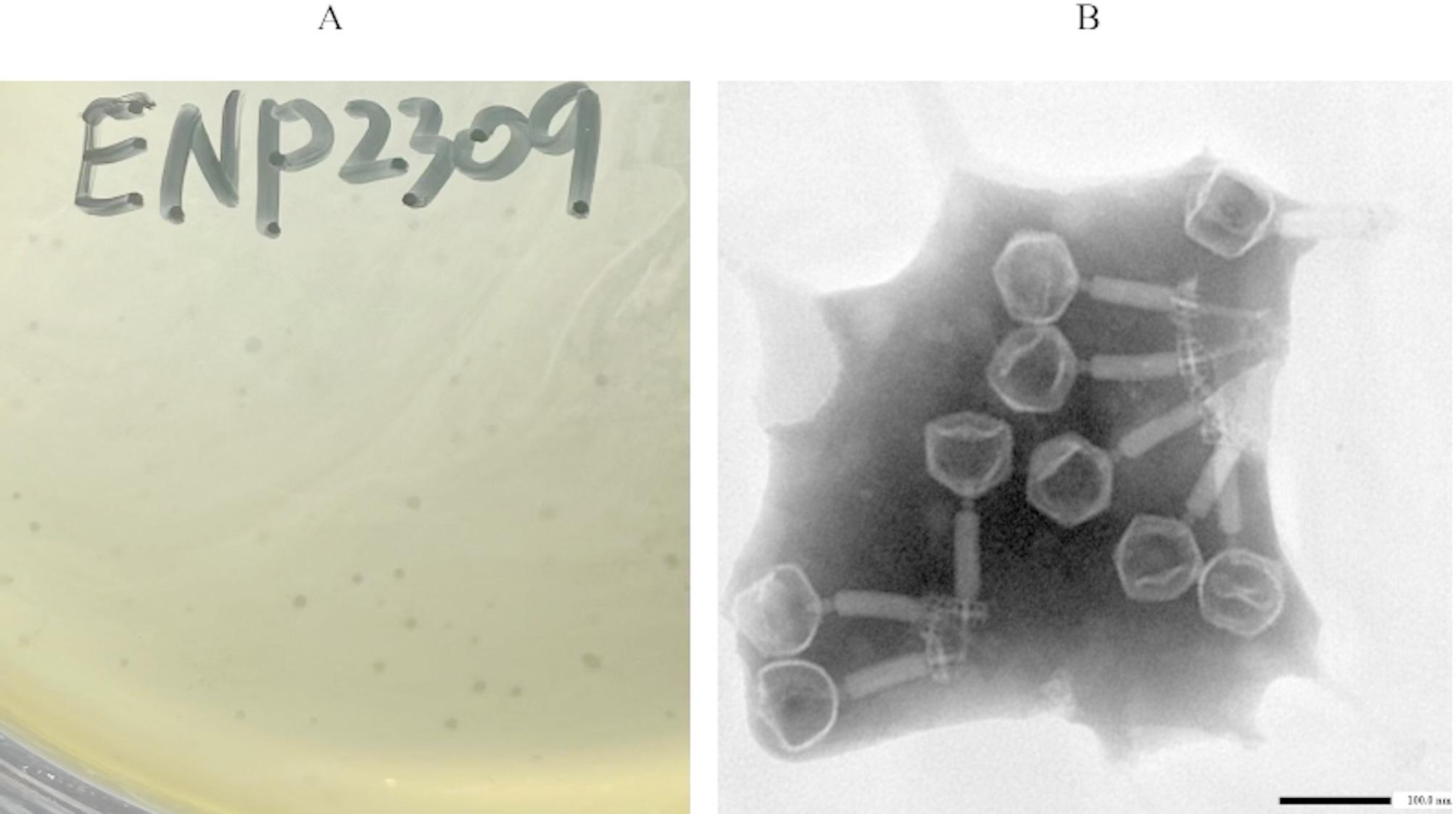
Morphology of Enterococcus phage ENP2309 (**A**) Plaque morphology of phage ENP2309 on double-layer agar plates; (**B**) transmission electron micrograph showing the phage structure of ENP2309

A bacteriophage targeting *Enterococcus* strains was isolated from sewage samples using the double-layer agar method. After three successive rounds of purification, a clonal phage population, exhibiting uniform plaque morphology was obtained (Fig. 1A). Transmission electron microscopy (TEM) revealed that phage ENP2309 features an icosahedral head (70 ± 1 nm in diameter) and a contractile tail (145 ± 2 nm in length) (Fig. 1B).

### Biological characteristics

**Fig. 2:**
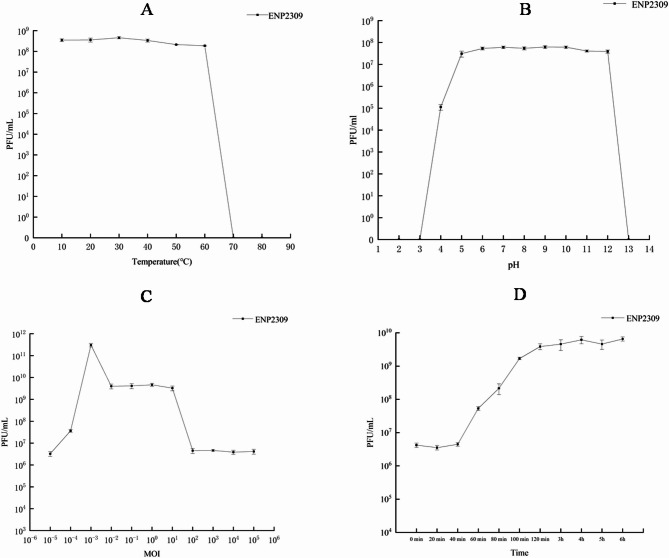
Biological characteristics of phage ENP2309

Host specificity profiling revealed that phage ENP2309 infected 13/19 (68.4%) *Enterococcus* isolates (Table 2), confirming its broad-spectrum lytic ability against diverse *E. faecalis*. strains. The phage ENP2309 exhibited stable lytic activity across a broad temperature range (10–60 °C) but was irreversibly inactivated at temperatures exceeding 70 °C (Fig. 2A); The phage ENP2309 demonstrated remarkable pH stability, maintaining infectivity between pH 3–12, although complete inactivation occurred at extreme pH conditions (≤ 3 or ≥ 12) (Fig. 2B); The phage ENP2309 attained a maximal concentration of 5.0 × 10¹¹ PFU/mL at an MOI of 0.001, establishing this as the optimal MOI for propagation (Fig. 2C); One-step growth analysis revealed that ENP2309 displayed a 40-minute latent period before entering a 120-minute burst phase (40–120 min postinfection), and the burst size was 920 PFU/cell (Fig. 2D).

**Table 2:** The host range of phage ENP2309

Number		Species	Strain	Origin	Plaque Morphology Scoring	EOP	Antibiotic Resistance
1	*E. faecalis*		GZ16185	Sheep	་4	0.66	ERY/CLI/VAN/TET
2	*E. faecalis*		GZ25	Sheep	་4	1.00	ERY/CLI/TET
3	*E. faecalis*		GZ85	Cattle	་4	0.65	ERY/CLI/TET
4	*E. faecalis*		GZ01	Cattle	་4	0.70	ERY/CLI/TET
5	*E. faecalis*		GZ39	Cattle	་4	0.62	ERY/CLI/TET
6	*E. faecalis*		GZ14008	Chicken	་4	0.69	ERY/CLI/TET
7	*E. faecalis*		S29	Chicken	་4	0.52	ERY/CLI/TET
8	*E. faecalis*		S38	Cattle	་1	1.23E-07	ERY/CLI/TET
9	*E. faecalis*		S46	Pig	་1	9.09E-08	ERY/CLI/TET
10	*E. faecalis*		S24	Sheep	་4	0.45	ERY/CLI/TET
11	*E. faecalis*		S42	Cattle	་4	0.78	ERY/CLI/TET
12	*E. faecalis*		D-1	Pig	་2	1.37E-07	CLI/AMP
13	*E. faecalis*		10–217	Chicken	་2	2.82E-07	ERY/CLI/TET/PEN/AMP
14	*E. gallinarum*		S23	Sheep	0	0.00	CLI
15	*E. faecalis*		S39	Pig	0	0.00	ERY/CLI/TET
16	*E. casseliflavus*		S26	Chicken	0	0.00	CLI/VAN/AMP
17	*E. faecalis*		S25	Sheep	0	0.00	ERY/CLI/VAN/TET
18	*E. faecalis*		S41	Cattle	0	0.00	ERY/CLI/TET/AMP
19	*E. faecium*		Y34	Chicken	0	0.00	AMP

Note: ERY stands for Erythromycin, CLI stands for Clindamycin, TET stands for Tetracycline, VAN stands for Vancomycin, PEN stands for Penicillin, and AMP stands for Ampicillin

### Genomic characterization of the bacteriophage ENP2309

The complete genome of phage ENP2309 (GenBank accession: PQ787220) comprises a linear double-stranded DNA molecule of 148,806 bp, with the genomic features summarized in Table 3. Among the 153 predicted genes, 67 open reading frames (ORFs) were functionally annotated on the basis of homology to characterized genes, whereas the remaining 86 ORFs encoded hypothetical proteins of unknown function.

**Fig. 3:**
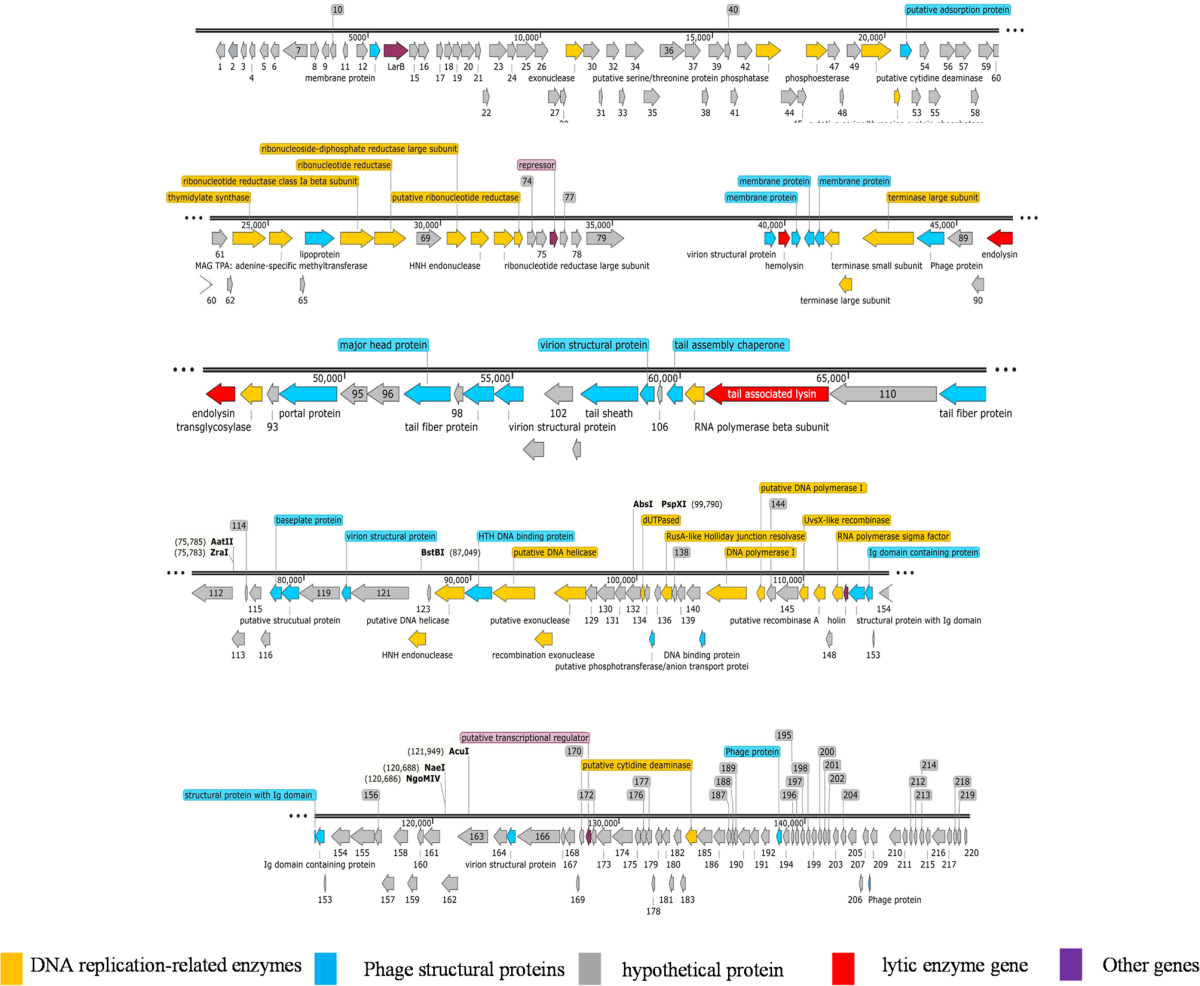
The annotated genome of phage ENP2309

Annotated ORFs are categorized into three major functional groups: (a) structural proteins (such as membrane proteins, virion structural proteins, major head proteins, and tail fiber proteins); (b) DNA replication/repair machinery proteins (putative DNA helicases, DNA primases, and DNA polymerases); and (c) lytic enzymes (such as hemolysin, endolysin, and tail-associated lysins) (Fig. 3). Notably, the genome lacks hallmark lysogeny-associated genes (such as integrases, excisionases, or repressors), confirming that phage ENP2309 can be classified as a strictly virulent phage. The genome encodes eight tRNA genes corresponding to the following amino acids: aspartate (Asp), tryptophan (Trp), arginine (Arg), leucine (Leu), proline (Pro), methionine (Met), tyrosine (Tyr), and threonine (Thr).

**Table 3:** Basic characteristics of the bacteriophage ENP2309 genome

Phage	Genome size bp	GC percent %	The number of ORFs	The total length of ORFs (bp)	The average length of ORFs bp	The percentage of ORFs %
ENP2309	148 806	35.90	220	130 209	591.86	87.50

**Fig. 4:**
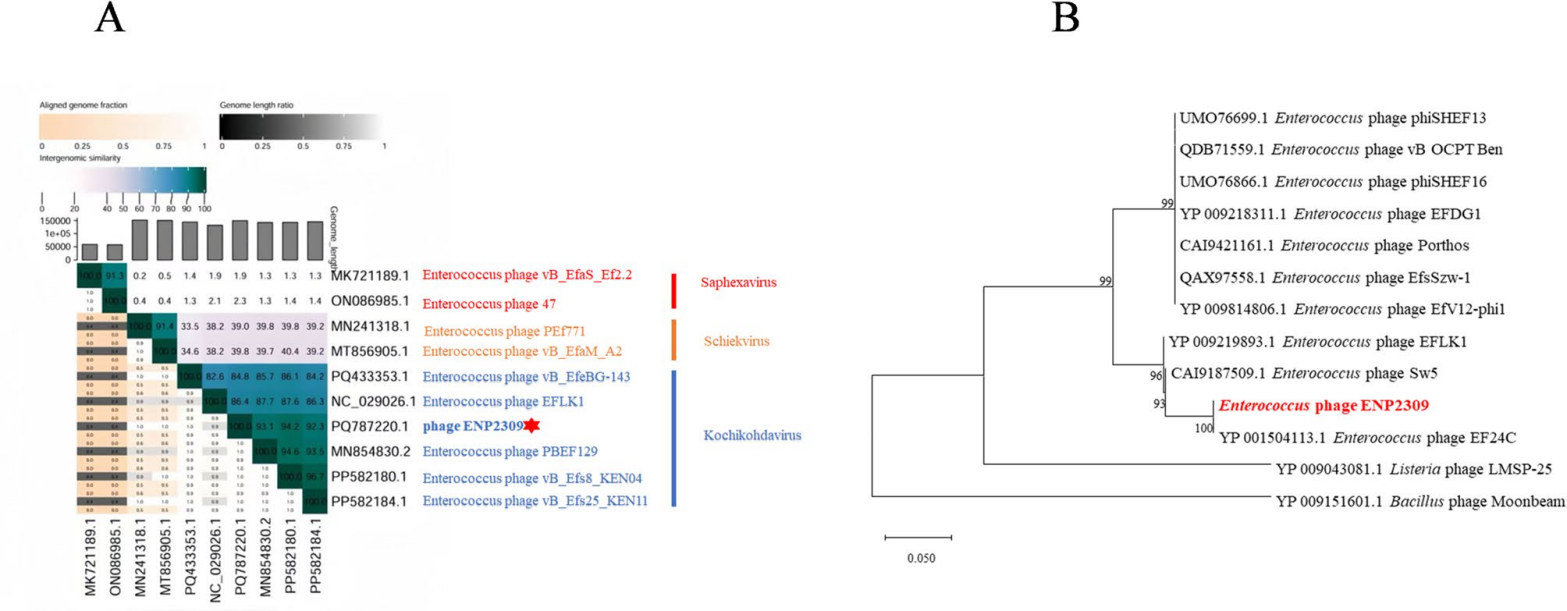
Phylogenetic relationships of phage ENP2309 (**A**) Genome similarity comparison of phage ENP2309 with other phages; (**B**) Phylogenetic tree based on the large terminase subunit

Phylogenetic analysis revealed that the bacteriophage ENP2309 exhibited the closest evolutionary clustering with Enterococcus phage EFLK1 (GenBank: NC_029026.1) and Enterococcus phage PBEF129 (GenBank: MN854830.2), both of which are members of *Kochikohdavirus*. Enterococcus phage EFLK1 (isolated in Israel, 2014) possesses a 130,952 bp genome encoding 198 protein-coding genes [20], whereas phage PBEF129 features a larger 141,520 bp genome with 241 predicted genes [21]. The shared phylogenetic lineage strongly supported the taxonomic classification of ENP2309 within *Kochikohdavirus* (Fig. 4A). Comparative analysis of the large terminase subunit (TerL) further supported the phylogenetic placement of ENP2309 within *Kochikohdavirus*, showing high amino acid identity to the Enterococcus phage EF24C (GenBank: YP_001504113.1) (Fig. 4B).

### Evaluation of treatment efficacy

#### Physical condition, survival rate and body weight

**Fig. 5:**
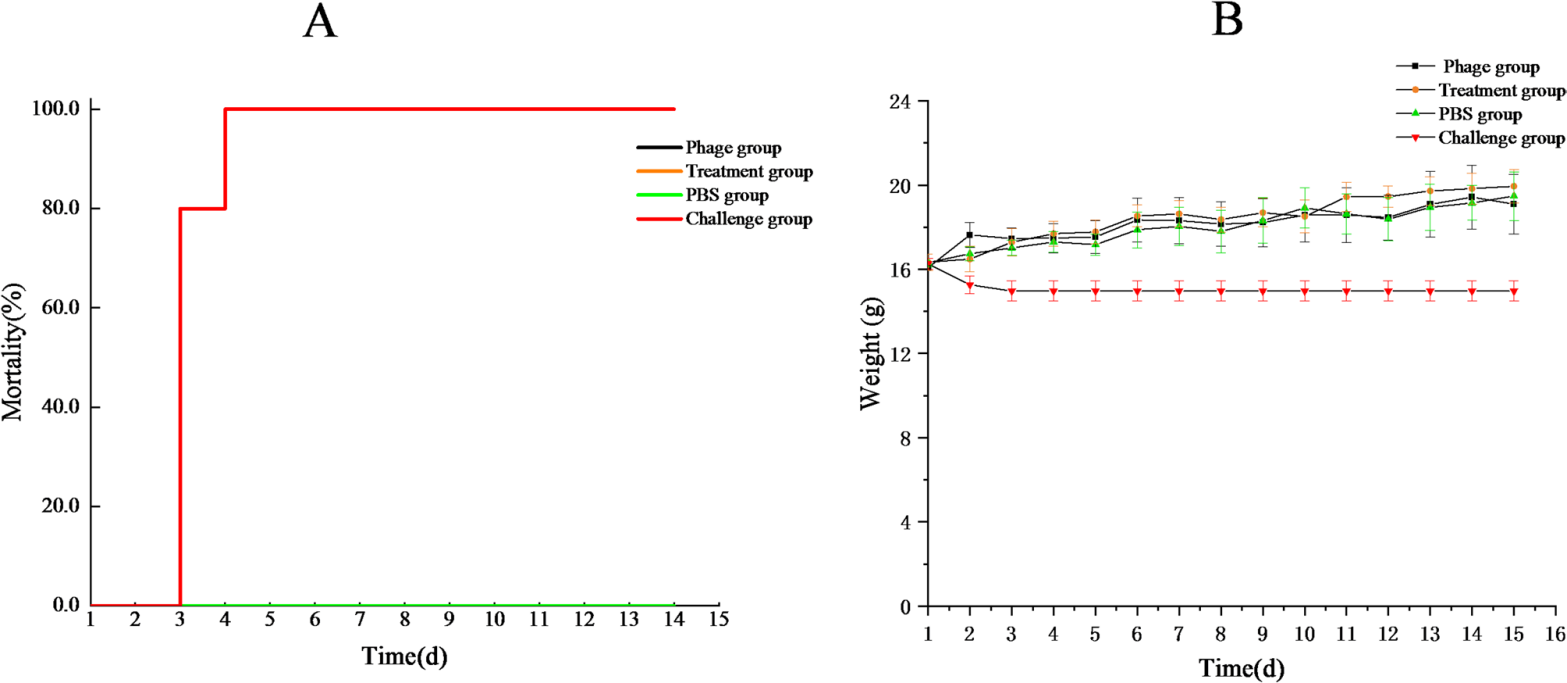
Mortality rates and weight changes in mice (**A**) mortality; rate and (**B**) body weight

Clinical assessments on Day 2 post-infection revealed profound intergroup differences: the challenge group presented characteristic disease manifestations (unkempt fur, ocular discharge, perianal diarrhoea, and severe lethargy), whereas the treatment, phage, and PBS groups maintained normal physical conditions without pathology. The treatment group, phage group and PBS group presented 0 deaths (0%, 0/10) during the 14-day monitoring period, whereas the challenge group presented uniform mortality with all the mice succumbing to infection within 96 h after the challenge (Fig. 5A). Body weight was initially uniform (16–18 g), followed by divergent patterns: the challenge group experienced progressive wasting until death, whereas the treatment group displayed only transient 24 h weight loss before complete recovery and resumption of normal growth, paralleling the stable gains in the PBS groups (Fig. 5B). These findings collectively demonstrate the dual ability of ENP2309 to rescue infected hosts while maintaining an exemplary safety profile for clinical translation.

#### Pathological changes in visual organs

**Fig. 6:**
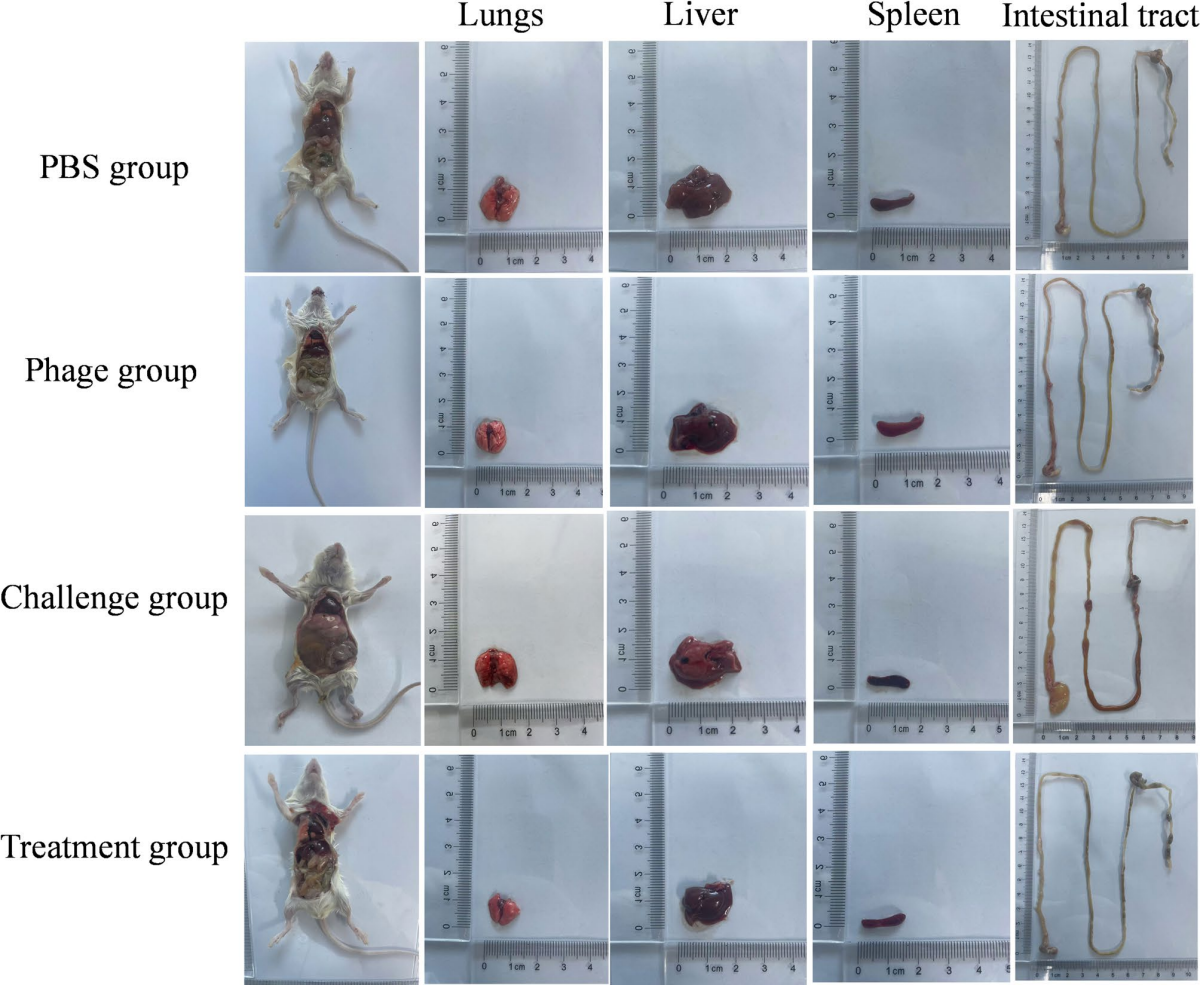
Observation of pathological changes in organs

Histopathological analysis revealed extensive enteric pathology characterized by severe diarrhea-associated lesions throughout the intestinal tract, including significant intestinal wall thinning, marked mucosal oedema, and diffuse luminal swelling, concurrently, extraintestinal organs (liver, lung, and spleen) exhibited significant pathological changes, including congestion, bleeding and swelling in the challenge group. In a contrast, phage ENP2309 treatment resulted in nearly-complete histological recovery of all affected organs (lung, liver, spleen, and intestinal tract), with tissue architecture restoration comparable to that of the PBS group (Fig. 6).

#### Major organ lesions

**Fig. 7:**
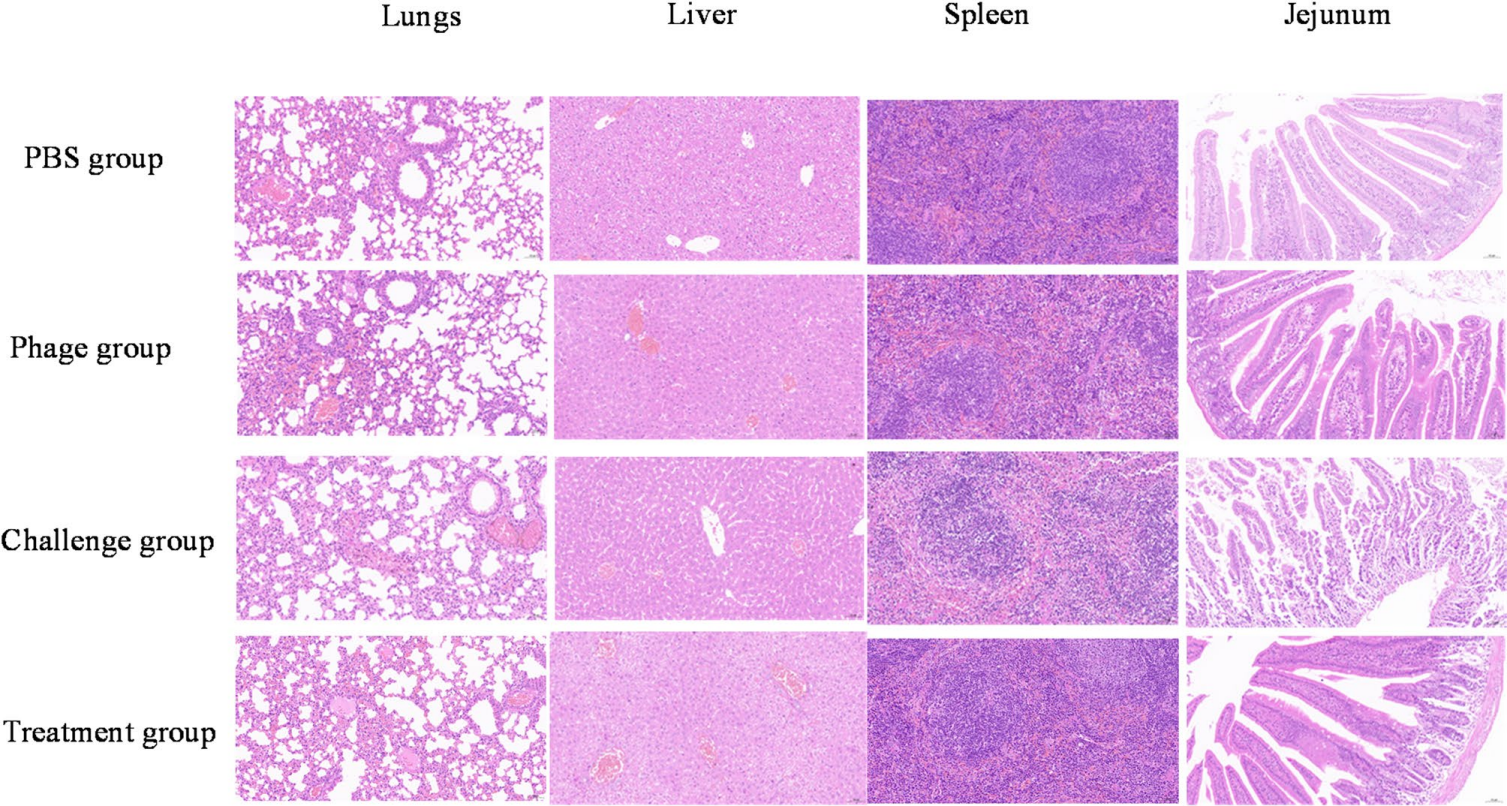
Pathological changes in major organs

Histopathological examination revealed distinct organ-specific pathological manifestations in the challenge group: infected mice exhibited marked hepatic damage characterized by obscured lobular boundaries, hepatocyte oedema, and cytoplasmic vacuolization; pulmonary alterations including focal alveolar wall thickening, septal widening, and minimal cellular necrosis; and severe intestinal pathology featuring mucosal architecture disruption, extensive villous epithelial detachment, and loss of interlayer demarcation. In contrast, the treatment, phage, and PBS groups presented only minor alveolar wall thickening with otherwise normal organ histology (Fig. 7). These findings demonstrate: that multiple organs are involved in *E. faecalis* infection, that the intestinal epithelium is the primary pathological target, and that organ integrity is preserved in phage-treated mice.

#### Bacterial load in organs

**Fig. 8:**
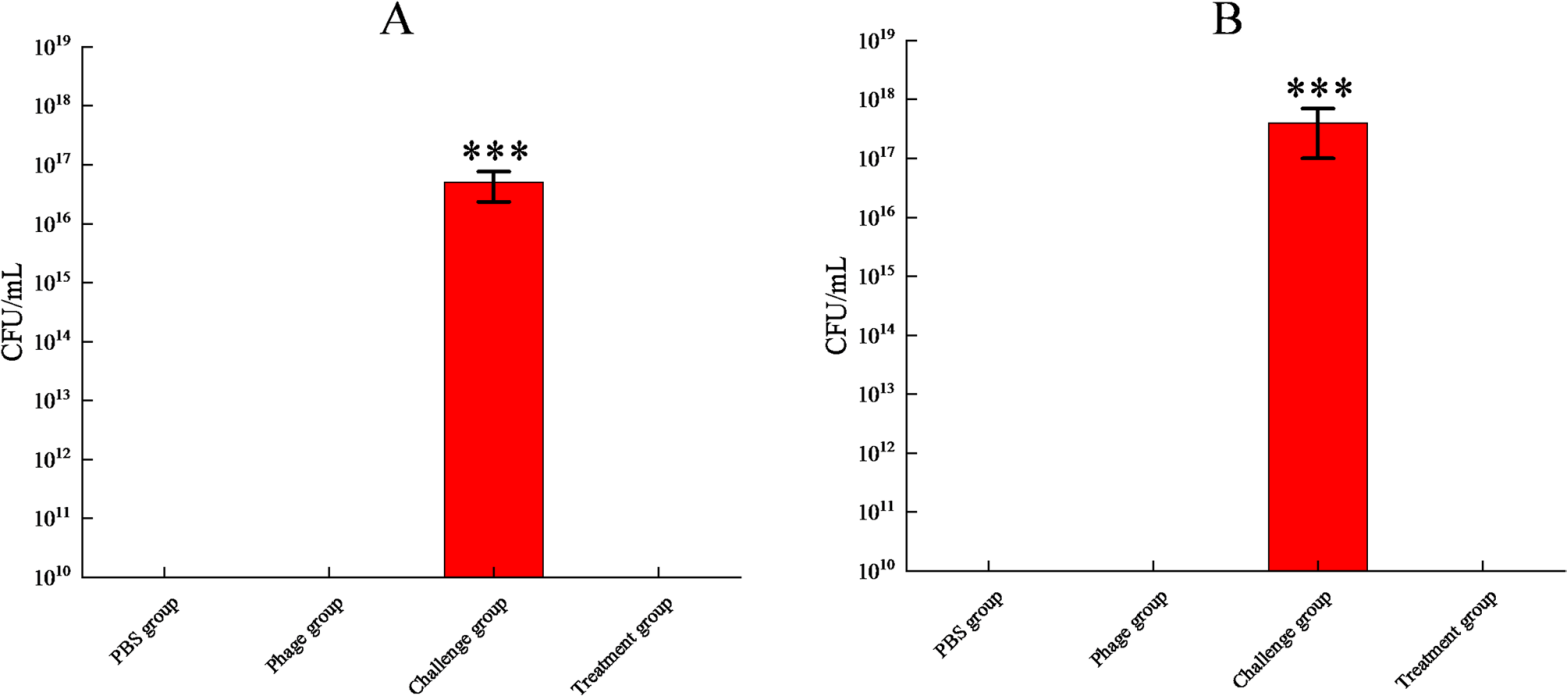
Bacterial load of organs. The challenge group was measured after the death of the mice, whereas the other groups were measured at the end of one treatment cycle (after 14 days). (**A**) Liver; (**B**) spleen

Quantitative analysis after the 14-day treatment period revealed complete bacterial clearance in the spleen and liver of phage-treated mice (0 CFU/g tissue), which was significantly lower (*p* < 0.001) than that in the heavily colonized challenge group, whereas both the phage control and PBS groups presented the expected baseline sterility (Fig. 8), confirming the therapeutic efficacy of ENP2309 in eradicating systemic infection without inducing bacterial dissemination in control mice.

#### Cytokine dynamics

**Fig. 9:**
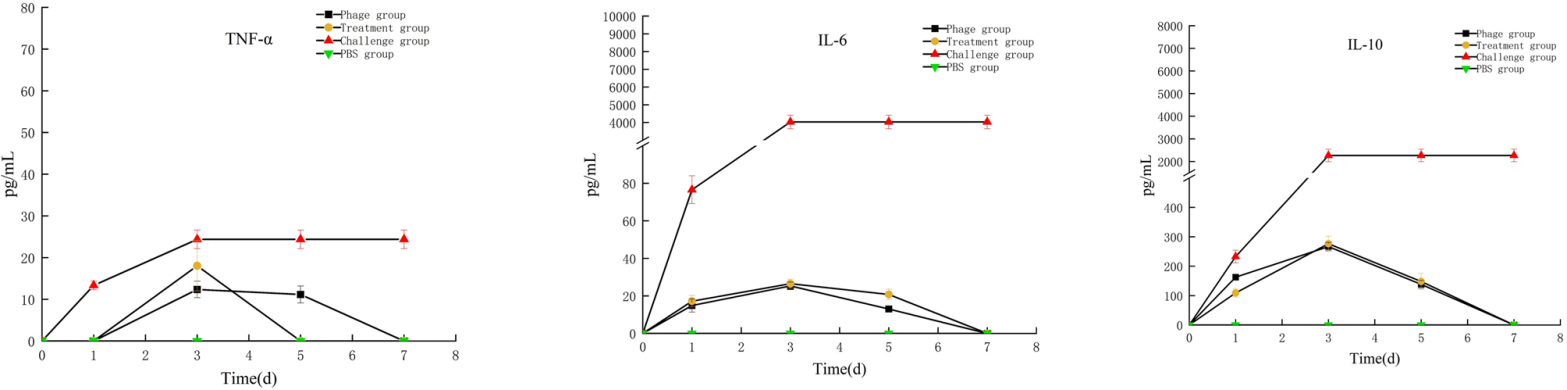
Cytokine monitoring in mice peripheral blood

Dynamic cytokine profiling revealed distinct temporal patterns across the experimental groups (Fig. 9): the PBS group maintained physiological levels throughout the experimental period; the challenge group exhibited dramatic increases in the levels of proinflammatory cytokines including IL-6, IL-10 and TNF-α, peaking at Day 3 post-infection; and the treatment and phage groups demonstrated a unique biphasic regulatory pattern— an early phase (0–3 days) of controlled immune activation induced by phage, followed by a late phase (3–7 days) of rapid inflammatory resolution through negative feedback mechanisms, ultimately returning to control levels by Day 7. These kinetic characteristics confirm that the phage operates through a three-stage mechanism of “rapid bactericidal action-inflammatory regulation-homeostasis restoration”, which not only effectively controls pathogen load but also precisely maintains immune balance through regulatory mechanisms, thereby providing an innovative therapeutic strategy combining potent antibacterial activity with immunomodulatory function for the clinical management of multidrug-resistant *Enterococcus* infections.

## Discussion

The global AMR crisis, which claims millions of lives annually [3], has underscored the limitations of conventional antibiotics—lengthy development cycles and diminishing efficacy against rapidly evolving superbugs [22]. This urgent unmet need has revitalized interest in bacteriophage therapy, with successful isolation methods targeting critical pathogens such as *Klebsiella pneumoniae* [23–24], *Acinetobacter baumannii* [25], *Pseudomonas aeruginosa* [26], *Escherichia coli* [27–28], and others [29–34]. In addition to their proven clinical efficacy against multidrug-resistant pathogen infections [35–37], phages offer versatile applications, including food safety [38–39], diagnostics [40], and biofilm disruption [41]. Notably, Gram-positive-targeting phages present distinct manufacturing advantages by circumventing endotoxin purification—a major production hurdle—thereby streamlining development and reducing costs. These attributes position phage therapy as a multifaceted solution in the postantibiotic era.

This study aimed to isolate and characterize a novel lytic broad-spectrum phage targeting VRE strains, with subsequent validation through integrated morphological characterization, biological profiling, and genomic analysis to confirm its clinical applicability, followed by mice intraperitoneal challenge models demonstrating 100% survival efficacy. Distinct from previously reported short-tailed enterococcal phages vB_ZEFP and VE18393 epaX [42–43], the phage ENP2309 exhibits superior bioproduction efficiency, achieving maximal yields of 5.0 × 10¹¹ PFU/mL at an exceptionally low optimal MOI (0.001 versus conventional phages’ 0.01 ~ 0.1) [42–43]. Furthermore, compared with phagesEF24C, Phi_Eg_SY1, and vB_EfaS-Zip, ENP2309 has enhanced thermal resilience [20, 44], coupled with advantageous replication kinetics — a 40-minute latency period enabling faster host lysis than phage vB_EfmH_163, but achieving a 5.9-fold greater burst size than phage vB_EfmH_163 [45–46].

The complete genome of the bacteriophage ENP2309 indeed encodes one gene that could be considered deleterious: hemolysin [47]. While hemolysins encoded by certain bacterial genomes are known to cause erythrocyte lysis [48], it is unknown whether a hemolysin encoded by a phage genome would similarly affect red blood cells. Consequently, we meticulously designed our study to include a phage-only control group. The primary purpose of establishing this control group was to rigorously assess whether this particular gene might exert any adverse effects on somatic cells. We evaluated this potential impact on the host by assessing parameters such as mice body weight, survival rate, and histopathological analysis. Furthermore, no hemolysis was observed in the serum samples collected from the mice during cytokine level monitoring. On the basis of these comprehensive observations, we concluded that, despite the integration of this gene into the phage genome, we did not observe significant hemolytic activity or adverse effects associated with this gene. Nevertheless, given the inherent complexity of gene‒environment interactions, this issue warrants rigorous scrutiny. Prior to clinical translation, comprehensive safety assessments must be implemented to fully evaluate potential health impacts—including functional validation through isogenic knockout constructs and expanded cohort sizes— to substantiate the findings’ reliability.

While phages have demonstrated significant in vitro antibacterial activity, their clinical translation requires rigorous in vivo validation. In this study, we evaluated the therapeutic potential of the bacteriophage ENP2309 in a BALB/c mice model of VRE infection. Notably, a single administration of ENP2309 achieved 100% protection, outperforming the efficacy of phage vB_EfaH_163 reported in comparable studies [46] and circumventing the potential therapeutic limitations associated with repeated dosing-induced immune responses [49–50]. Comprehensive safety assessments revealed no significant differences in weight, survival rate, histopathology, or peripheral cytokine levels between the treatment and PBS groups, although transient cytokine elevation was observed, which is consistent with previous findings [51]. Most notably, our findings demonstrated complete microbial clearance of *E. faecalis* from hepatic and splenic tissues, in stark contrast to the challenge group, which sustained high-grade bacteremia alongside marked histopathological injury.

Furthermore, we observed that the bacterial load in the spleen was greater than that in the liver in the challenge group. This may be attributed to the following factors: (1) The spleen is a crucial organ for blood filtration, and is responsible for clearing aged red blood cells, foreign substances, and pathogens from the bloodstream. When bacteria enter circulation—for example, rapidly following intraperitoneal injection—the spleen captures these bacteria, leading to a higher bacterial load. (2) The spleen is rich in immune cells, such as macrophages and lymphocytes, which actively phagocytose and eliminate bacteria. However, when bacterial numbers are substantial, these immune cells may also serve as “reservoirs” for bacteria, resulting in bacterial accumulation within the spleen [52].

These results demonstrate that the phage ENP2309 exhibits critical characteristics of an ideal therapeutic agent: such as robust lytic efficacy against VRE, a safe profile, and superior biological characteristics compared with previously reported phages, thereby establishing it as a promising antimicrobial candidate for treating VRE infections.

However, the use of a single phage alone is likely to promote the emergence of resistant bacterial strains [53–54]. In the future, we will comprehensively investigate the resistance mechanisms triggered by a single phage.

## Conclusions

In this study, we isolated a broad-host-range phage, ENP2309, which efficiently lysed VRE strains. The phage ENP2309 exhibited strong therapeutic efficacy, providing 100% protection to VRE infected mice with a single dose, suggesting its potential as a therapeutic agent for managing VRE infections.

## Data Availability

No datasets were generated or analysed during the current study.

## Abbreviations

AMP: Ampicillin
Arg: Arginine
Asp: Aspartate
bp: Base Pairs
CFU: Colony-Forming Unit
CLI: Clindamycin
ERY: Erythromycin
EOP: Efficacy of Plating
GC: Guanine-Cytosine content
HE: Hematoxylin-Eosin
Leu: Leucine
MDR: Multi-Drug Resistance
Met: Methionine
MOI: Multiplicity of Infection
NCBI: National Center for Biotechnology Information
OD: Optical Density
ORF: Open Reading Frame
PBS: Phosphate Buffered Saline
PEN: Penicillin
PFU: Plaque-Forming Unit
Pro: Proline
TEM: Transmission Electron Microscopy
TET: Tetracycline
THB: Todd-Hewitt Broth
Thr: Threonine
Trp: Tryptophan
Tyr: Tyrosine
VAN: Vancomycin
VRE: Vancomycin-Resistant Enterococcus
WHO: World Health Organization
